# Proteomic analysis of nipple aspirate fluid from women with early-stage breast cancer using isotope-coded affinity tags and tandem mass spectrometry reveals differential expression of vitamin D binding protein

**DOI:** 10.1186/1471-2407-6-68

**Published:** 2006-03-16

**Authors:** Timothy M Pawlik, David H Hawke, Yanna Liu, Savitri Krishnamurthy, Herbert Fritsche, Kelly K Hunt, Henry M Kuerer

**Affiliations:** 1 Department of Surgical Oncology, The University of Texas M. D. Anderson Cancer Center, Houston, Texas, USA; 2 Department of Molecular Pathology, The University of Texas M. D. Anderson Cancer Center, Houston, Texas, USA; 3 Department of Pathology, The University of Texas M. D. Anderson Cancer Center, Houston, Texas, USA; 4 Department of Laboratory Medicine, The University of Texas M. D. Anderson Cancer Center, Houston, Texas, USA

## Abstract

**Background:**

Isotope-coded affinity tag (ICAT) tandem mass spectrometry (MS) allows for qualitative and quantitative analysis of paired protein samples. We sought to determine whether ICAT technology could quantify and identify differential expression of tumor-specific proteins in nipple aspirate fluid (NAF) from the tumor-bearing and contralateral disease-free breasts of patients with unilateral early-stage breast cancer.

**Methods:**

Paired NAF samples from 18 women with stage I or II unilateral invasive breast carcinoma and 4 healthy volunteers were analyzed using ICAT labeling, sodium dodecyl sulfate-polyacrylamide gel (SDS-PAGE), liquid chromatography, and MS. Proteins were identified by sequence database analysis. Western blot analysis of NAF from an independent sample set from 12 women (8 with early-stage breast cancer and 4 healthy volunteers) was also performed.

**Results:**

353 peptides were identified from tandem mass spectra and matched to peptide sequences in the National Center for Biotechnology Information database. Equal numbers of peptides were up- versus down-regulated. Alpha2HS-glycoprotein [Heavy:Light (H:L) ratio 0.63] was underexpressed in NAF from tumor-bearing breasts, while lipophilin B (H:L ratio 1.42), beta-globin (H:L ratio 1.98), hemopexin (H:L ratio 1.73), and vitamin D-binding protein precursor (H:L ratio 1.82) were overexpressed. Western blot analysis of pooled samples of NAF from healthy volunteers versus NAF from women with breast cancer confirmed the overexpression of vitamin D-binding protein in tumor-bearing breasts.

**Conclusion:**

ICAT tandem MS was able to identify and quantify differences in specific protein expression between NAF samples from tumor-bearing and disease-free breasts. Proteomic screening techniques using ICAT and NAF may be used to find markers for diagnosis of breast cancer.

## Background

Despite the widespread adoption of screening mammography, many breast cancers still escape detection at an early stage [1,2]. Identifying relevant biologic markers could improve our ability to diagnose early-stage breast cancer [3-7]. Compared with DNA analysis (genomics) and RNA analysis (expression profiling), the examination of protein expression (proteomics) may be more relevant to tumor physiology as protein stability and activity are largely what determine cellular phenotype and function [8]. Recent developments in proteomics have enabled high-throughput analysis of thousands of proteins, making possible the identification of new biologic markers [9-11].

One of the most useful techniques that has emerged for the detection of proteins and protein-derived peptides is mass spectrometry (MS) [12-14]. Using surface-enhanced laser desorption ionization time-of-flight (SELDI-TOF) MS, we previously found differences in the phenotypic proteomic profiles of nipple aspirate fluid (NAF) samples from patients with early-stage breast cancer versus healthy female volunteers [15]. However, although MS is powerful and allows, in principle, for the detection of many copurifying proteins in a fraction, it remains difficult with MS to distinguish specific from nonspecific interactions and to detect quantitative changes in protein complex abundance and composition without direct visualization of the proteins in gels [16-18]. This is mainly because profiling experiments such as SELDI in which MS-1 only is performed, and not MS/MS, is not an inherently quantitative technique [16,19-22] and does not allow for the specific identification of individual peptides.

The development of methods and instrumentation for automated, data-dependent electrospray ionization MS in conjunction with microcapillary liquid chromatography and database searching has significantly increased the sensitivity and speed of large-scale protein identification directly from mixtures [19,23,24]. Recently developed isotope-coded affinity tag (ICAT) technology has greatly expanded the range of proteins that can be analyzed, quantified, and identified using these techniques [19,25,26]. Unlike two-dimensional polyacrylamide gel electrophoresis (PAGE) and SELDI-TOF MS, which comparatively profile the naturally occurring forms of peptides and proteins, ICAT analysis profiles the relative amounts of cysteine-containing peptides derived from tryptic digests of protein extracts [9]. The isotope tags [Cys-(na) light and Cys-(9 ^13^C) heavy] covalently bind to cysteine moieties of amino acids within proteins [9,26,27]. ICAT has a large dynamic range, and the two isotope labels act as mutual internal standards for quantitation [28]. The mass difference between the proteins labeled with the light and heavy tags allows for the separation and comparison of peptides from each sample set [19,26]. The small fraction of proteins lacking cysteine is transparent to analysis, and only relative changes in protein abundance are interrogated [28]. After the peptide mixture is separated using a reverse-phase liquid chromatography electrospray ionization mass spectrometer, the peptides are identified by sequence database analysis [16]. In this manner, ICAT proteomic analysis allows for not only identification but also quantification of differentially expressed proteins.

The purpose of the current study was to analyze NAF from women with early stage breast cancer using quantitative proteomic ICATs and tandem MS (MS/MS). In this paper, we show that ICAT tandem MS is able both to identify and quantify differences in specific protein expression between NAF samples from tumor-bearing and disease-free breasts. Our findings have important implications as they suggest that proteomic screening techniques using ICAT and NAF may be used to find markers for diagnosis of breast cancer.

## Methods

### Patients and sample collection

NAF samples were obtained from 18 women with stage I or II unilateral invasive breast carcinoma who presented to The University of Texas M. D. Anderson Cancer Center's Nellie B. Connally Breast Center. NAF was also collected from 4 healthy volunteers not related to the patients. Individuals were eligible to participate if they had not previously undergone subareolar surgery, which might have disrupted the terminal ductal system. All participants gave written informed consent to undergo bilateral nipple aspiration. The study protocol was approved by M. D. Anderson Cancer Center's institutional review board.

Before aspiration was attempted, the nipple was initially cleansed with a small amount of Omniprep paste (D.O. Weaver and Co., Aurora, CO) to remove any keratin plugs and then wiped with an alcohol pad. A small amount of lotion was placed on the breast, and the breast was gently massaged from the chest wall toward the nipple for 1 minute. Ductal fluid was collected as previously described by Sartorius et al. [29]. In brief, nipple aspiration was accomplished using a handheld suction cup (Product Health, Inc., Menlo Park, CA) similar to the nonpowered breast pumps commonly used to express milk from lactating women. With the suction cup over the nipple, the plunger of the syringe was withdrawn to the 5- to 10-mL level until ductal fluid was visualized. The fluid droplets were collected into a 10-μl graduated micropipette (Drummond Scientific Co., Broomall, PA). In the case of women with breast cancer, NAF was collected separately from the tumor-bearing and disease-free breasts. The volumes of NAF were recorded for each patient and each breast.

Immediately after collection, the NAF samples were rinsed into centrifuge tubes containing sterile phosphate-buffered saline supplemented with the protease inhibitors 4-(2-aminoethyl)-benzenesulfonylfluoride HCl (0.2 mM), leupeptin (50 μg/mL), aprotinin (2 μg/mL), and dithiothreitol (0.5 mM). The samples were then centrifuged at 1500 RPM for 10 minutes, and the supernatants were collected and suspended in a 500 μl PBS buffer with the previously mentioned protease inhibitors.

### ICAT analysis

**Figure 1:**
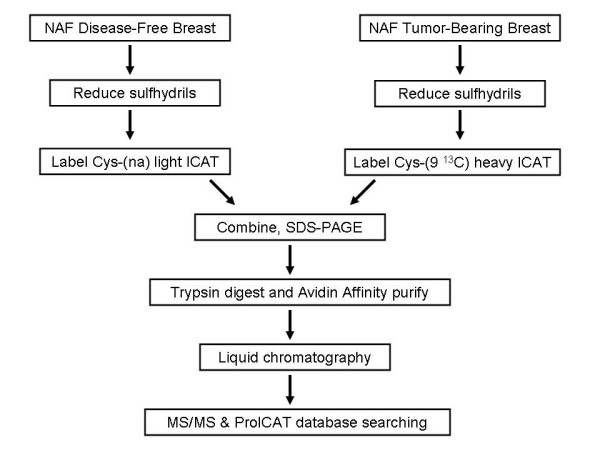
Schema of ICAT analysis performed using a Cleavable ICAT Reagent Kit (Applied Biosystems, Foster City, CA) according to the manufacturer's guidelines, with some modifications. Avidin Affinity refers to the Avidin Affinity Cartridge kit (Applied Biosystems, Foster City, CA)

ICAT analysis was performed using a Cleavable ICAT Reagent Kit (Applied Biosystems, Foster City, CA) according to the manufacturer's guidelines with some modifications (Figure 1) [30]. For ICAT analysis, the NAF protein samples from the tumor-bearing breasts and disease-free breasts of patients with early-stage breast cancer were respectively pooled in a normalized fashion. One hundred micrograms of each NAF protein pool was separately reduced in 80 μL of denaturing buffer at 37°C for 2 hours with 5 mM TCEP and alkylated at 37°C for 2 hours in the dark with ICAT-heavy (NAF from tumor-bearing breasts) and ICAT-light (NAF from disease-free breasts) reagent. After alkylation, ICAT-heavy and ICAT-light reactions were mixed together and concentrated by centrifugal evaporation, SDS-PAGE loading buffer was added, and the sample was separated by SDS-PAGE. The gel was cut into 14 slices and subjected to in-gel trypsin digestion at 37°C for 20 hours. After extraction, the ICAT-labeled peptides were purified using the ICAT Avidin Buffer Pack and Avidin Affinity Cartridge kit (Foster City, CA) according to the manufacturer's guidelines. Briefly, the peptide mixture was dried by vacuum centrifugation and resolubilized in the loading buffer of the kit. The peptide mixture was loaded in the Avidin Affinity Cartridge and washed twice with two kinds of wash buffer (first wash in 2× PBS followed by a second wash in 50 mM NH_4_HCO_3 _in 20% MeOH) to reduce the salt concentration and remove nonspecifically bound peptides. The ICAT-labeled peptides were then eluted with the elution buffer and dried by vacuum centrifugation. The dried peptides were cleaved from the biotin portion of the ICAT reagent with the cleaving reagents at 37°C for 2 hours. The ICAT-labeled peptides were then dried by vacuum centrifugation and resolubilized in 2% formic acid for liquid chromatography and MS.

### Liquid chromatography and mass spectrometry

Liquid chromatography and MS were performed using an LCPackings nano-LC system consisting of an Ultimate pump, Famos autosampler, and Switchos column-switching device for on-line desalting (Dionex Corp., Sunnyvale, CA). The Switchos was fitted with a C18 reversed phase column (Pepmap C18, 300 μmIDx1 mm,) and a C18 reversed phase nanocolumn (Pepmap C18, 75 μmIDx10 mm). Reversed phase solvents were 0.005% heptafluorobutyric acid in either water and 2% acetonitrile or 80% acetonitrile. Gradient elutions were performed over 90 minutes from about 5% acetonitrile to about 40% followed by flushing with 72% acetonitrile and reequilibration for the next injection. Spectra were acquired on a QqTOF quadrupole/time-of-flight mass spectrometer (Qstar Pulsar-I, Applied Biosystems/MDS Sciex, Foster City, CA). Qstar was externally calibrated, using the doubly and triply charged peaks of the peptide neurotensin. One survey MS scan was followed by two MS/MS scans on the two most intense ions from the MS spectrum, omitting previously selected targets.

### Database searches

The acquired MS/MS spectra were automatically compared against the National Center for Biotechnology Information (NCBI) database using the ProICAT program (Applied Biosystems/MDS Sciex) allowing 1 missed cleavage (trypsin) and 0.3 and 0.5 d windows for MS/MS and MS resp. Modifications allowed were fairly typical: ICAT on Cys, Met oxidation. The same program was also used to calculate ICAT ratios. The ICAT experiment by design produced fewer peptides, but the requirement of containing Cys increases the confidence of the one peptide hits when manually curated (as these were). We extracted tables of high-confidence (99%) matched peptides with their ratios and used them for further calculations. The quality of peptide matches of interest was checked manually.

### Western blot analysis

Protein samples (40 μg) were separated on a 10% SDS-PAGE gel and transferred (170 V for 45 minutes) to nitrocellulose filters (pure nitrocellulose; pore size, 0.45 μm; Bio-Rad, CA). Blots were blocked overnight at 4°C in 5% (wt/vol) skim milk-TTBS (0.1% Tween 20-Tris-buffered saline). The next morning the membranes were incubated for 1 hour in 5% skim milk in PBS with polyclonal anti-vitamin D-binding protein (Biodesign International, Saco, ME) diluted 1:1000. Membranes were then incubated with goat anti-rabbit secondary antibody (Santa Cruz, CA) for 1 hour at 4°C. The bound antibody was visualized using the ECL-Plus Detection kit (Amersham Pharmacia Biotech, Piscataway, NJ) and the bands were analyzed by video imaging and densitometry.

### Statistical analyses

Densitometry measurements were analyzed and statistical significance was determined using independent Student's t-test. A standard cut-off of 15–20% change is commonly utilized to denote differential expression. In the current experiments, a peptide ratio of 1.56 correlated with one standard deviation above the median ratio.

## Results

The median age for the 18 patients with stage I or II unilateral invasive breast carcinoma was 48 years. The majority of the patients (73%) were premenopausal. The median age of the volunteers was 55 years. All 4 healthy volunteers were postmenopausal.

### ICAT analysis of NAF

**Figure 2:**
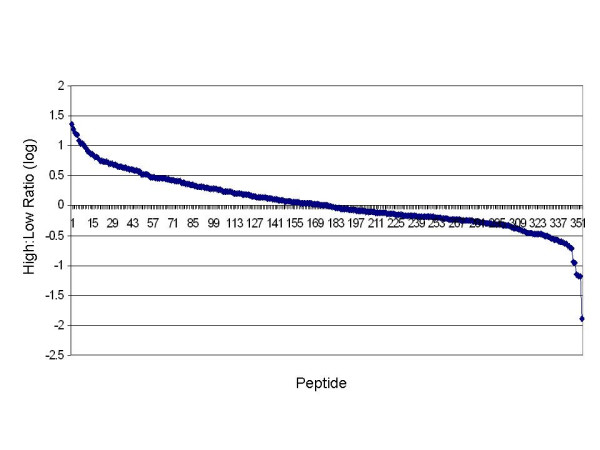
Using the ProICAT bioinformatics program, 353 peptides were identified. Approximately equal numbers of proteins were up- versus down-regulated.

NAF from the tumor-bearing breasts versus disease-free breasts of 10 patients with unilateral early stage breast cancer was assessed by ICAT analysis. Using the ProICAT bioinformatics program, 353 peptides were identified with a high level of confidence (> 99%) on the basis of matching of tandem mass spectra of peptides to peptide sequences in the NCBI database. Approximately equal numbers of peptides were up- versus down-regulated (Figure 2). Following manual review of all peptide match spectra and exclusion of redundant matches, 39 proteins were identified that had sufficient quality to justify assignment (Table 1). Twenty proteins (51%) were identified based on two or more peptide matches; 19 proteins (49%) were identified based on a single peptide match.

As previously noted, peptides from tumor-bearing breasts had a heavy tag, and peptides from disease-free breasts had a light tag. Of the 39 proteins, 19 had an ICAT ratio of Heavy:Light (H:L) > 1.0, 16 proteins had an ICAT ratio of H:L < 1.0, and 1 protein had an ICAT ratio of H:L = 1.0. In three cases, a peptide signal could only be convincingly detected in one, not both states (diseased versus not), so no ratio could be calculated. Proteins that were differentially expressed between the NAF from tumor-bearing breasts and the NAF from disease-free breasts included, among others, alpha2HS-glycoprotein (H:L ratio 0.63), lipophilin B (H:L ratio 1.42), beta-globin (H:L ratio 1.98), hemopexin (H:L ratio 1.73), and vitamin D-binding protein precursor (H:L ratio 1.82) (Table 1). For the one-peptide hit of interest, Vitamin-D binding protein, a second peptide, not containing Cys was identified using Mascot (data not shown).

**Figure 3:**
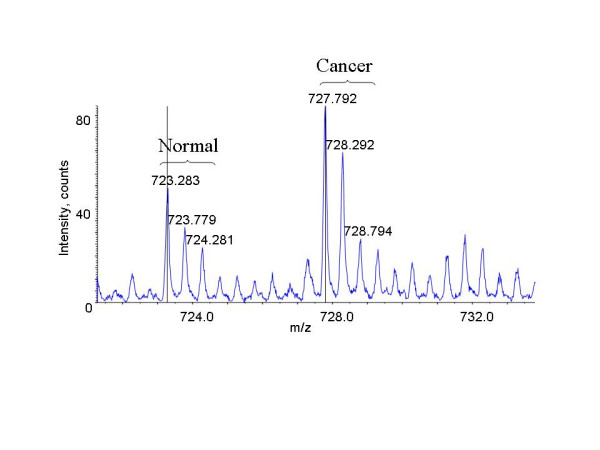
Quantification of vitamin D-binding protein through the ICAT-labeled parent ion signals of the peptide VC*SQYAAYGEK.

**Figure 4:**
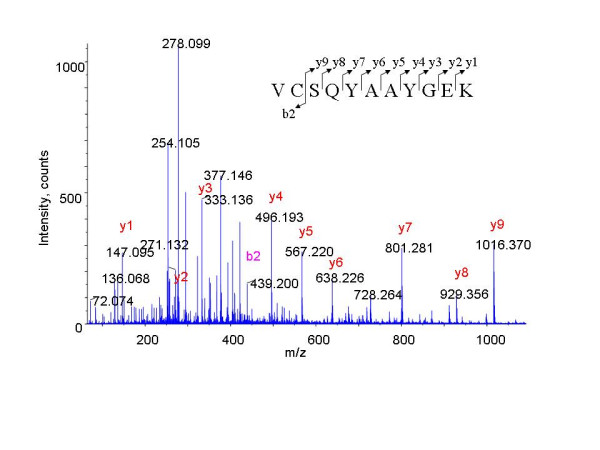
Identification of the peptide VC*SQYAAYGEK from vitamin D-binding protein by MS/MS.

The finding that ICAT identified vitamin D-binding protein as being overexpressed in NAF from the tumor-bearing breasts of women with early-stage breast cancer was particularly interesting (Figures 3 and 4). Previous studies [31-34] have suggested that the vitamin D receptor may be overexpressed in breast tumors. We therefore sought to examine further the expression of vitamin D-binding protein in a separate, independent set of NAF specimens to validate the ICAT vitamin D findings.

### Western blot analysis

**Figure 5:**
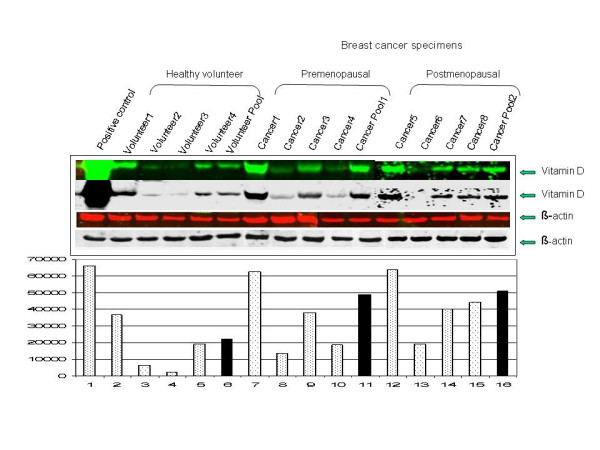
Western blot analysis of independent NAF sample set. The relative difference in vitamin D-binding protein expression in the pooled samples of NAF from healthy volunteers versus NAF from women with early-stage breast cancer was two-fold (*P *= 0.04; standard error = 9703.7 (solid bars). Individual NAF specimens showed variation in vitamin D-binding protein expression among both healthy volunteers and women with early-stage breast cancer (dotted bars). Menopausal status did not affect expression of vitamin D-binding protein in NAF.

To assess the correspondence of the ICAT vitamin D-binding protein data with an independent assay of protein abundance, we performed Western blot analysis on NAF obtained from a separate cohort of 12 women (4 healthy volunteers and 8 women with early-stage breast cancer). On western blot analysis, vitamin D-binding protein expression in the pooled samples of NAF specimens from women with early-stage breast cancer was twice that in the NAF specimens from healthy volunteers (*P *= 0.04; standard error = 9703.7) (Figure 5). Interestingly, the relative difference in expression was similar to the abundance ratio derived from ICAT analysis (1.82). Although the data derived from ICAT and pooled western analyses were similar, western blot analysis of individual NAF specimens showed variation in vitamin D-binding protein expression among both healthy volunteers and women with early-stage breast cancer (Figure 5). Menopausal status did not affect expression of vitamin D-binding protein expression in NAF from women with early-stage breast cancer (*P *= 0.57; standard error = 14425.3).

## Discussion

To our knowledge, the current study is the first proteomic analysis of women with breast cancer using ICAT tandem MS and NAF. NAF was chosen as a substrate to evaluate biomarkers of breast disease because nipple aspiration is simple, quick, reliable, and reproducible [13-15,35,36]. In addition, nipple aspiration is completely noninvasive and requires a device with only minimal cost. Nipple aspiration provides concentrated secreted proteins not diluted with the irrigation fluid that is required to perform procedures such as ductoscopy or ductal lavage. Because of this, NAF is a rich source of proteins [37], thereby making it a good potential candidate as a source of biomarkers for early diagnosis of breast cancer [35,37-40].

In the current study, ICAT identified 39 proteins that were differentially expressed in tumor-bearing versus disease-free breasts. Among the proteins identified, alpha2HS-glycoprotein was underexpressed, while lipophilin B, beta-globin, hemopexin, and vitamin D-binding protein precursor were overexpressed. A number of other proteins were identified, however these identifications had lower ProICAT scores. Despite being confirmed with manual curation, one may need to use Mascot or other secondary filters to eliminate the chance of false positive identification.

Alpha2HS-glycoprotein is important in blocking transforming growth factor-beta1 signal transduction, which is associated with tumor progression and resistance to chemotherapy in established cancers [41,42]. Alpha2HS-glycoprotein has been observed to be depleted in certain tumors compared with normal tissue [42], consistent with the decreased ICAT ratio reported here.

Overexpression of lipophilin B, beta-globin, and hemopexin has also been confirmed in tumor-bearing tissues using non-ICAT techniques. Specifically, lipophilin B mRNA is overexpressed in 70% of breast tumors, and serum antibodies to lipophilin B have been detected in breast cancer patients [43-45]. Similarly, beta-globin has been found to be overexpressed in a number of different cancers compared with normal tissue [46-48]. Hemopexin, a protein involved in matrix metalloproteinase (MMPs) activation, has been linked to invasion and metastasis in many cancer model systems, including human breast cancer [49]. MMP activation requires an intact hemopexin domain, and overexpression of hemopexin may increase MMP activity, degradation of extracellular matrix, and tumor cell migration [50,51].

Our finding that vitamin D-binding protein precursor was overexpressed in the tumor-bearing breasts of women with breast cancer also agrees with previous findings. Receptors for 1,25-dihydroxyvitamin D have been shown to exist in cultured breast cancer cells and in primary breast cancers [32-34,52]. In fact, the vitamin D-receptor has been reported to be present in 80% of breast cancers [32] and to be expressed at significantly higher concentrations in breast cancers than in normal breast tissue [31].

ICAT technology has been successfully used to study protein expression in yeast [16,19], plant [53], and mammalian hematopoietic [25,54] and liver cells [55]. A review of the available literature reveals, however, that ICAT technology has not been employed in the clinical investigation of breast cancer. To date, only one study [56] has utilized stable isotope proteomic analysis to investigate ductal carcinoma of the breast. Zang et al. [56] compared protein expression between 10,000 cells each of microdissected normal ductal epithelium and metastatic ducal carcinoma using MS/MS in combination with ^16^O/^18^O isotopic labeling introduced enzymatically. A total of 76 proteins were identified, and some of the proteins – such as mitochondrial isocitrate dehydrogenase, actin, and 14-3-3 protein xi/delta – were found to be significantly upregulated in breast tumor cells [56]. Although similar to ICAT, the stable isotope technique used in these experiments did not employ an affinity component to enhance the separation of the labeled peptides.

ICAT technology is still in the early phase of development and should be regarded as a screening step to identify potentially interesting protein changes, which need to be independently confirmed by immunoblotting before they can be considered confirmed [25]. In the current study, we performed western blot analysis on independent NAF samples to confirm the overexpression of vitamin D-binding protein that had been noted with ICAT. Due to limited sample material, immunoblotting could not be performed on the other proteins identified by ICAT. Vitamin D-binding protein was chosen because of its known association with breast cancer and because it had a high ICAT ratio (1.82), indicating significantly different expression in the tumor-bearing breast versus disease-free breasts. Western blot analysis confirmed that, in general, vitamin D-binding protein was significantly overexpressed in NAF obtained from women with breast cancer, although there was variation in expression among patients. Comparison of abundance ratios measured by ICAT and by Western blotting of the *pooled *specimens showed similar values (i.e., a two-fold increase in vitamin D-binding protein in the NAF from the tumor-bearing versus disease-free breasts). Previous reports have confirmed that the ICAT method is highly accurate for the quantification of proteins. The ICAT method has accurately predicted the relative quantification of proteins at known concentrations in mixtures [16,19,57,58]. Comparison of abundance ratios obtained by ICAT and by Western blot analysis has also consistently shown similar values [59]. In aggregate, these data strongly suggest that the ICAT method can be used to accurately measure the changes in abundance of differentially expressed proteins.

Reports in the literature indicate that ICAT technology can identify over 100 proteins per sample [9]. The relatively small number of proteins identified in the current study was most likely related to the preponderance of a few highly abundant, but non-specific, proteins in NAF. Similar to serum and plasma, in which about 75% of the total protein is comprised of albumin or immunoglobulins, NAF has high levels of albumin, lactotransferrin, immunoglobulins, and apolipoprotein D. In the future, we intend to remove these proteins prior to ICAT analysis to facilitate the MS identification of other, potentially more relevant proteins. Newer stable-isotope technology such as the iTRAQ system (Applied Biosystems), which allows for the direct comparison of up to four samples in a single experiment rather than the two samples possible with ICAT, may be informative. Use of this strategy would allow the direct inclusion of two additional clinically relevant samples – such as NAF from women with ductal carcinoma in situ and NAF from women with fibrocystic disease.

## Conclusion

Proteomics has the potential to be a powerful tool in identifying molecules to assist in the diagnosis and treatment of breast cancer. In fact, Somiari et al. [9] and others [7] have argued that breast cancer prognostication and management will be significantly improved only by the use of multiple biomarkers, which most likely will be identified by proteomic analysis of breast carcinoma. Comprehensive analysis of proteomic profiles derived from biologic tissues and fluids remains, however, a formidable task. Complex protein mixtures must be resolved into individual proteins or manageable groups of proteins, quantified, and then identified. ICAT technology enables not only qualitative but also quantitative analysis of normal and diseased specimens, making identification of cancer-specific protein markers possible. The quantitative proteomic strategy of ICAT and NAF utilized in the current study is an example of a novel approach to discovering biomarkers in women with breast cancer.

## List of abbreviations

ICAT: Isotope-coded affinity tag

NAF: nipple aspirate fluid

SDS-PAGE: sodium dodecyl sulfate-polyacrylamide gel

MS: mass spectrometry

MS/MS: tandem mass spectrometry

SELDI-TOF: surface-enhanced laser desorption ionization time-of-flight

H:L: heavy:light

NCBI: National Center for Biotechnology Information (NCBI)

## Competing interests

The author(s) declare that they have no competing interests.

## Authors' contributions

TMP, DHK, YL, KKH, and HMK made substantial contributions to conception, design, analysis, acquisition, interpretation of the data, as well as drafting and approving the final manuscript. SK and HF made substantial contributions to conception and interpretation of the data, critical revisions of the manuscript and final approval of the manuscript.

## Pre-publication history

The pre-publication history for this paper can be accessed here:
